# The effect of exercise intensity types on the self-rated health status of young-old comorbidities patients: a cross-sectional study in Guangdong, China

**DOI:** 10.3389/fpubh.2023.1292712

**Published:** 2023-11-16

**Authors:** Linjin Li, Fengfeiyue Dai, Dan Zhang

**Affiliations:** Tsinghua Shenzhen International Graduate School, Institute for Hospital Management, Tsinghua University, Shenzhen, China

**Keywords:** young-old, older comorbidities, chronic disease, physical activity, exercise intensity, self-rated health

## Abstract

**Objective:**

Explore the effect of different types of exercise intensity on the self-rated health status of young-old comorbid patients with cardiovascular disease and metabolic disease, as well as the differences in effect among different genders. Provide more references and suggestions for chronic disease management in older comorbidities patients based on the results of the study.

**Methods:**

A multi-stage stratified cluster random sampling method was used to select older (≥60 years old) comorbidities patients from communities in Guangdong Province as the survey subjects. Using the “Survey Questionnaire on the Current Status and Influencing Factors of older Comorbidities Patients,” face-to-face interviews were conducted with 1,300 old patients. Data from 965 young-old patients (aged 60–74) who simultaneously suffered from cardiovascular and metabolic diseases were selected. Unordered multifactor Logistic regression analysis was used to explore the association between the type of exercise intensity and self-rated health. Stratified analysis was performed based on gender.

**Results:**

The results of unordered multivariate logistic regression analysis showed that compared with young-old comorbidities patients with poor self-rated of health status, young-old comorbidities patients who underwent vigorous-intensity exercise were more likely to have better self-rated of health status (OR = 4.368, 95% CI: 2.491–7.661, *p* < 0.05). Stratified analysis based on gender showed that for young-old comorbidities male patients, those who engaged in vigorous-intensity exercise were more likely to have better self-rated of health status (OR = 2.924, 95% CI = 1.266–6.751, *p* < 0.05); for young-old comorbidities female patients, those who were encouraged by their family to exercise (OR = 2.460, 95% CI: 1.143–5.291, *p* < 0.05), participate in social activities (OR = 6.173, 95% CI: 2.285–16.678, *p* < 0.05), and engage in vigorous-intensity (OR = 4.232, 95% CI: 1.869–9.583, *p* < 0.05) or moderate-intensity exercise (OR = 4.555, 95% CI: 1.825–11.368, *p* < 0.05) were more likely to have better self-rated of health status.

**Conclusion:**

If the physical condition allows, vigorous-intensity exercise has a significant positive effect on the self-rated of health status of young-old comorbidities patients with cardiovascular disease and metabolic disease. Specifically, for young-old comorbidities male patients, those who engage in vigorous-intensity exercise are more likely to self-evaluate their health as good; for young-old comorbidities female patients, both vigorous-intensity and moderate-intensity exercise can improve their self-rated of health status.

## 1. Introduction

As residents’ life expectancy increases, the disease spectrum is changing. According to the recent research by the British Medical Journal (BMJ), aging is currently the main factor causing comorbidities in high-income countries around the world, and the proportion of people with two or more diseases is continuously increasing (1). WHO defined that comorbidities refer to the simultaneous presence of two or more chronic diseases in an individual (2). Older comorbidities refer to people aged 60 and above who suffer from comorbidities and are the main group of chronic comorbidities (3). The probability of comorbidities have exceeded the probability of getting one single chronic disease (4); in the study by Zhang et al., it was found that among the middle-aged and older population aged 50 and above in China, the number of comorbidities patients is 2.4 times that of patients with one single chronic disease (5). Compared to suffering from one single chronic disease, chronic comorbidities can significantly reduce the quality of life of older patients, cause more health losses, increase the economic burden on families, and consume more social medical resources. The issue of older comorbidities patients has become one of the main public health issues in China (and even the world), and it is a major challenge that countries must face in building an aging society (6). If there is a lack of scientific and efficient methods for the prevention and management of chronic comorbidities, it will increase the burden of medical services and affect the normal life of individuals, families, and society (7).

A study analyzed data from the 2018 China Health and Retirement Longitudinal Study (CHARLS) and found that the probability of chronic comorbidities among older people aged 60 and above in Guangdong Province were 43.7% (8); the research of Wang et al. found that the main chronic diseases were hypertension, chronic pain, inflammatory connective tissue disease, diabetes and dyslipidemia in Guangdong Province (9). The harm brought by chronic comorbidities to the older people is continuously accumulating, so the key to managing chronic comorbidities in older patients should be moved forward, and more attention should be paid to prevention and control compared to treatment and relief. Lack of exercise is an independent risk factor for chronic disease (10). In the chronic disease management guidelines of other countries, the importance of sports is emphasized, especially for cardiovascular and cerebrovascular diseases, diabetes and musculoskeletal diseases (11, 12), and different sports methods have different effects on the prevention/rehabilitation of chronic diseases. Therefore, identifying and analyzing the effect of exercise patterns or intensity on the health of older comorbidities patients has positive significance for optimizing clinical diagnosis and treatment guidelines for chronic comorbidities and health management of comorbidities patients (13).

Previous studies have explored the association between exercise and older people’ health from multiple perspectives such as the type and frequency of exercise (14, 15), and have shown that the health benefits of exercise vary depending on demographic characteristics (16). There have been studies that have shown gender differences in the association between exercise and health (17) and between health behavior and the onset of chronic diseases (18). Therefore, different guidance measures should be taken for male and female older comorbidities patients in preventing chronic disease or improving the quality of life of chronic disease patients through exercise behavior. However, there are currently few studies focusing on gender differences in the management of chronic diseases in young-old comorbidities patients with different exercise intensity. Therefore, in order to better understand the effect of different types of exercise on the health of older comorbidities patients of different genders, this study intended to use the data from “The current situation and influencing factors of older comorbidities in Guangdong Province” to explore the effect of different types of exercise intensity on the health status of older comorbidities patients. Further this article analyzed the different effects on male and female older comorbidities patients and explore the reasons. Suggestions were also provided for the practice of older chronic comorbidities patients’ management, promoting the health of older patients with chronic comorbidities.

## 2. Materials and methods

### 2.1. Data source and data sampling

The data comes from a questionnaire survey of older chronic comorbidities patients in communities in Guangdong Province. Based on the Health Status Survey Scale (SF-36) [Chinese Version (19)], Health Promotion Lifestyle Scale (HPLP-C) (20), and 8-item Morisky Questionnaire (MMAS-8) (21), a survey questionnaire on the current situation and influencing factors of older comorbidities was designed, which included socio-demographic data and disease-related data, including gender, age, marital status, residential status, education level, duration of illness, work status, per capita annual income of families, body mass index (BMI), number and types of comorbidities, exercise style, exercise frequency, self-rated of health status, lifestyle, and self-management ability. The effectiveness and feasibility of this questionnaire survey has been proven through Delphi expert consultation method and pre survey experiments. A questionnaire survey was conducted by interviewing patients with chronic comorbidities by community general practice clinics, centralized discussions among residents, hospital management graduate students, general practice standardized training interns, community general practitioners, and nurses who have undergone unified training as investigators.

This survey used the Guangdong Provincial Community Resident Health Record Information System and adopted a multi-stage stratified cluster random sampling method. In the first stage, three cities were randomly selected in Guangdong Province based on economic level and geographical location. In the second stage, three districts were randomly selected from each city. In the third stage, three communities were randomly selected from each district for investigation. After signing the informed consent, we investigated the patients aged over 60 and living in the community for more than 1 year, and have been diagnosed with two or more chronic diseases (mainly combined with hypertension, diabetes, hyperlipidemia, coronary heart disease, stroke, diabetes, chronic obstructive pulmonary disease, osteoporosis, osteoarthropathy and other common chronic diseases) by hospitals at the second level or above. A total of 1,300 older comorbidities patients were surveyed based on the proportion of older comorbidities patients in the city to the old population, according to the formula for calculating the sample size of a cross-sectional survey, N=μα/22P 21−P/δ2 (22), α = 0.05, and taking into account a 20% inefficiency. An effective questionnaire is defined as survey data without missing values and duplicate responses are removed. A total of 1,000 valid datasets were obtained, with a questionnaire effective recovery rate of 76.9%.

Previous studies have classified older people in the age group of 60–74 as young-old people (23). Most of these older people are in good physical condition and have the ability to take care of themselves. In contrast, they have better physical activity and responsiveness among the entire old population, and their acceptance and benefit from exercise are also relatively high. Therefore, this study sets the target user as the older people aged 60–74. According to the follow-up data of CHARLS in 2018 (the latest data released in September 2020), the prevalence of cardiovascular diseases (such as hypertension, dyslipidemia (high/low cholesterol), heart disease, stroke) and metabolic diseases (such as diabetes or elevated blood sugar) among older people with chronic comorbidity were relatively high (24), which indicated that these two types of diseases have a greater effect on the older patients with chronic comorbidity. Therefore, we selected 965 patients with both cardiovascular and metabolic diseases from the 1,000 young-old comorbidities patients effectively surveyed to continue the following study.

### 2.2. Outcome variables

Self-rated of health refers to a comprehensive evaluation of the health status of individuals based on their comprehensive physical, psychological, social, and role functions (25), which is simple and easy to implement. It allows users to understand the overall status of individuals and predict health-related mortality rates (26) by asking “What do you think of your health status.” Most researchers believe that self-rated health can reliably measure the health status and quality of life of older people (27), and previous studies have shown that self-rated health indicators have good validity among Chinese older people (28). When self-rated health indicators are used to estimate overall health status, explore factors influencing health, and determine overall health needs in research, biases may not have a substantial effect on the results. In large-scale social surveys, especially for older people, using self-rated health indicators to measure health status has an advantage that other measurement tools cannot match (25). Therefore, this article uses self-rated of health to measure the health level of older people.

In this study, we used the self-rated of health of the respondents as the outcome variable. Specifically, the response was measured using a 5-point Likert scale (29): 1 = very good, 2 = good, 3 = average, 4 = poor, and 5 = very poor. According to previous research (30, 31), we used a score of 3 as the cutoff point, and respondents who answered “1” or “2” were classified as good and assigned a value of 1; the respondents who answered “3” were still average and assigned a value of 2; respondents who answer “4” or “5” were classified as poor and assigned a value of 3.

### 2.3. Independent variables

In CHARLS, the definition of high-intensity exercise is that “vigorous activities make you breathe much harder than normal and may include heavy lifting, digging, plowing, aerobics, fast bicycling, and cycling with a heavy load.” The definition of moderate-intensity exercise is that “moderate physical activities make you breathe somewhat harder than normal and may include carrying light loads, bicycling at a regular pace, or mopping the floor.” The definition of low intensity exercise is that “low intensity exercise includes at work and at home, walking to travel from place to place, and any other walking that you might do solely for recreation, sport, exercise, or leisure.” Based on this, the present study combines the definitions of exercise modes and exercise frequency for older adults from the “Physical Activity Guidelines for the Chinese Population (2021)” (32) and the “Physical Activity Guidelines for Americans” (33), as well as the intensity classification from previous research (34). The classification of exercise intensity into five categories, namely sedentary activity, light-intensity activity, moderate-intensity activity, vigorous-intensity activity, and high-intensity activity, was adopted following the method proposed by Norton et al. (35). However, since the target population of this study is older people, sedentary activity and high-intensity activity are less likely to occur. Therefore, the final classification of exercise types includes light-intensity activity, moderate-intensity activity, and vigorous-intensity activity. The determination of the exercise type was based on the participants’ exercise methods and frequency.

The independent variable of this study is the type of exercise intensity. According to the previous literature (36), the control variables we included are gender, age, BMI, comorbidity duration, household registration type, education level, per capita annual income, living conditions, marital status, family members’ supervision on exercise, participation in social activities, taking health care products, and sleep quality. After conducting an overall analysis, we divided the respondents into two groups based on gender: male and female, and conducted separate analyses. The specific assignment of variables is shown in Table 1.

**Table 1:** Variables and assignments of independent variables.

Self-assessment of health status	Good = 1, Average = 2, Bad = 3
Exercise intensity type	Vigorous-intensity = 1, Moderate-intensity = 2, Light-intensity = 3
Variables	Assignment
Gender	Male = 1, Female = 2
Age	60–64 = 1, 65–69 = 2, 70–74 = 3
BMI	Low weight = 1, Normal weight = 2, Overweight = 3, Obesity = 4
Comorbidity duration	Less than 6 years = 1, 6–10 years = 2, More than 10 years = 3
Household registration type	Urban registration = 1, Rural registration = 2
Education level	Junior high school and below = 1, High school/Polytechnic school = 2, Junior college = 3, College and above = 4
Per capita annual income	Less than 30,000 yuan = 1, 30,000–50,000 yuan = 2, More than 50,000 yuan = 3
Living conditions	Living alone = 1, Living with spouse = 2, Living with children = 3
Marital status	Married = 1, Widowed = 2, Separated/Divorced = 3
Will family members supervise exercise	Will = 1, Will not = 2
Whether respondents participates in social activities	Yes = 1, No = 2
Does respondents take health products	Yes = 1, No = 2
Can respondents have at least 6 h of sleep per day	Yes = 1, No = 2

### 2.4. Statistical analysis

Since all included variables are categorical variables, the χ^2^-test was used to detect the correlation between each variable and self-rated health. Due to the parallel test, the result was *p* < 0.05. Therefore, an unordered multivariate logistic regression model was used to analyze the effect of exercise intensity types on the self-rated health of young-old patients with chronic comorbidities. The control variables with statistical significance in the χ^2^-test were input into the model using the forward stepping method. In the unordered multivariate logistic regression model, the dependent variable was the self-rated poor health as the control, and the independent variable is the one with the highest assigned value as the control. And a stratified and unordered multivariate logistic regression analysis was conducted on men and women based on gender. The data is represented as adjusted ORs (aORs) and their corresponding 95% confidence intervals. SPSS 27.0 software was used for statistical analysis, and *p* < 0.05 was used as the statistically significant difference.

## 3. Results

### 3.1. Characteristics of sampling respondents

Table 2 shows the characteristics of young-old patients with chronic comorbidities related to self-rated health (*n* = 965). Overall, 406 (42.07%) older patients with chronic comorbidities engaged in vigorous-intensity exercise, 175 (18.14%) engaged in moderate-intensity exercise, and 384 (39.79%) engaged in light-intensity exercise; 365 (37.82%) young-old patients with chronic comorbidities reported good self-rated health status, 472 (48.92%) reported average self-rated health status, and 128 (13.26%) reported poor self-rated health status. Specifically, there was a statistically significant difference (*p* < 0.05) in the self-rated health status of different genders, BMI, comorbidity duration, household registration types, per capita annual income, living conditions, marital status, family members’ supervision on exercise, participation in social activities, taking health care products, sleep qualities and exercise intensity types.

**Table 2:** Comparison of self-rated health of young-old comorbidities patients with different data.

Variables	*N*	Good self-rated health [*n*(%)]	Average self-rated health [*n*(%)]	Poor self-rated health [n(%)]		*p-*value
Exercise intensity type					63.384	<0.001
Vigorous-intensity	406	207(51.0)	173(42.6)	26(6.4)		
Moderate-intensity	175	54(30.9)	90(51.4)	31(17.7)		
Light-intensity	384	104(27.1)	209(54.4)	71(18.5)		
Gender					29.488	<0.001
Male	496	152(30.6)	284(57.3)	60(12.1)		
Female	469	213(45.4)	188(40.1)	68(14.5)		
Age					5.784	0.216
60–64	365	145(39.7)	174(47.7)	46(12.6)		
65–69	513	188(36.6)	261(50.9)	64(12.5)		
70–74	67	29(36.8)	28(42.5)	10(20.7)		
BMI					28.077	<0.001
Low weight	55	14(25.5)	32(58.2)	9(16.4)		
Normal weight	610	206(33.8)	321(52.6)	83(80.9)		
Overweight	246	126(51.2)	90(36.6)	30(12.2)		
Obesity	54	19(35.2)	29(53.7)	6(11.1)		
Comorbidity duration					47.822	<0.001
Less than 6 years	372	112(30.1)	224(60.2)	36(9.7)		
6–10 years	438	208(47.5)	166(37.9)	64(14.6)		
More than 10 years	155	45(37.6)	82(36.3)	28(18.1)		
Household registration types					8.849	0.012
Urban registration	798	318(39.8)	381(47.7)	99(12.4)		
Rural registration	167	47(28.1)	91(54.5)	29(17.4)		
Education level					12.019	0.062
Junior high school and below	211	71(33.6)	103(48.8)	37(17.5)		
High school/Polytechnic school	292	108(37.0)	155(53.1)	29(9.9)		
Junior college	242	89(36.8)	116(47.9)	37(15.3)		
College and above	220	97(44.1)	98(44.5)	25(11.4)		
Per capita annual income					12.938	0.012
Less than 30,000 yuan	164	46(28.0)	91(55.5)	27(16.5)		
30,000–50,000 yuan	341	137(40.2)	171(50.1)	33(9.7)		
More than 50,000 yuan	460	182(39.6)	210(45.7)	68(14.8)		
Living conditions					29.049	<0.001
Living alone	53	12(22.6)	27(50.9)	14(26.4)		
Living with spouse	733	308(42.0)	335(45.7)	90(12.3)		
Living with children	179	45(25.1)	110(61.5)	24(13.4)		
Marital status					17.854	0.001
Married	910	352(38.7)	446(49.0)	112(12.3)		
Widowed	52	11(21.2)	25(48.1)	16(30.8)		
Separated/Divorced	3	2(66.7)	1(33.3)	0(0.0)		
Will family members supervise exercise					17.398	<0.001
Yes	742	286(38.5)	376(50.7)	80(10.8)		
No	223	79(35.4)	96(43.0)	48(21.5)		
Whether respondents participates in social activities					29.696	<0.001
Yes	832	337(40.5)	401(48.2)	94(11.3)		
No	133	28(21.1)	71(53.4)	34(25.6)		
Does respondents take health products					51.531	<0.001
Yes	232	54(23.3)	119(51.3)	59(25.4)		
No	733	311(42.4)	353(48.2)	69(9.4)		
Can respondents have at least 6 h of sleep per day					42.552	<0.001
Yes	419	202(49.3)	172(42.0)	36(8.8)		
No	370	163(29.4)	300(54.1)	92(16.6)		

After grouping the data by gender, Table 3 shows that out of 496 young-old male patients with chronic comorbidities, 172 (34.67%) engaged in vigorous-intensity exercise, 96 (19.36%) engaged in moderate-intensity exercise, and 228 (45.97%) engaged in light-intensity exercise; 152 (30.64%) reported good health status, 284 (57.26%) reported average health status, and 60 (12.10%) reported poor health status. There were statistically significant differences (*p* < 0.05) in the self-rated health status of different ages, BMI, comorbidity duration, household registration types, living conditions, marital status, family members’ supervision on exercise, taking health care products, and exercise intensity types. Table 4 shows that out of 469 young-old female patients with chronic comorbidities, 234 (49.89%) engaged in vigorous-intensity exercise, 79 (16.85%) engaged in moderate-intensity exercise, and 156 (33.26%) engaged in light-intensity exercise; 213 (45.41%) reported good health status, 188 (40.09%) reported average health status, and 68 (14.50%) reported poor health status. There were statistically significant differences (*p* < 0.05) in the self-rated health status of different BMI, comorbidity duration, household registration types, per capita annual income, living conditions, family members’ supervision on exercise, participation in social activities, taking health care products, sleeping qualities, and exercise intensity types.

**Table 3:** Comparison of self-rated health of young-old comorbidities male patients with different data.

Variables	*N*	Good self-rated health [*n*(%)]	Average self-rated health [*n*(%)]	Poor self-rated health [n(%)]		*P-*value
Exercise intensity type					22.924	<0.001
Vigorous-intensity	172	75(43.6)	84(48.8)	13(7.6)		
Moderate-intensity	96	21(21.9)	58(60.4)	17(17.7)		
Light-intensity	228	56(24.6)	142(62.3)	30(13.2)		
Age					20.403	<0.001
60–64	169	60(35.5)	95(56.2)	14(8.3)		
65–69	278	83(29.9)	164(59.0)	31(11.2)		
70–74	49	9(18.4)	25(51.0)	15(30.6)		
BMI					15.823	0.012
Low weight	28	8(28.6)	14(50.0)	6(21.4)		
Normal weight	297	81(27.3)	184(62.0)	32(10.8)		
Overweight	138	57(41.3)	63(45.7)	18(13.0)		
Obesity	33	6(18.2)	23(69.7)	4(12.1)		
Comorbidity duration					33.742	<0.001
Less than 6 years	211	59(28.0)	135(64.0)	17(8.1)		
6–10 years	188	78(41.5)	91(48.4)	19(10.1)		
More than 10 years	97	15(15.5)	58(59.8)	24(24.7)		
Household registration types					17.510	0.004
Urban registration	389	123(31.6)	222(57.1)	44(11.3)		
Rural registration	107	29(27.1)	62(57.9)	16(15.0)		
Education level					12.019	0.062
Junior high school and below	87	25(28.7)	45(51.7)	17(19.5)		
High school/Polytechnic school	152	34(22.4)	103(67.8)	15(9.9)		
Junior college	151	50(33.1)	81(53.6)	20(13.2)		
College and above	106	43(40.6)	55(51.9)	8(7.5)		
Per capita annual income					3.170	0.531
Less than 30,000 yuan	86	22(25.6)	51(59.3)	13(15.1)		
30,000–50,000 yuan	180	60(33.3)	103(57.2)	17(9.4)		
More than 50,000 yuan	230	70(30.4)	130(56.5)	30(13.0)		
Living conditions					19.534	0.001
Living alone	40	7(17.5)	21(52.5)	12(30.0)		
Living with spouse	360	125(34.7)	196(54.4)	39(10.8)		
Living with children	96	20(20.8)	67(69.8)	9(9.4)		
Marital status					11.556	0.008
Married	459	144(31.4)	266(58.0)	49(10.7)		
Widowed	35	7(20.0)	17(48.6)	11(31.4)		
Separated/Divorced	2	1(50.0)	1(50.0)	0(0.0)		
Will family members supervise exercise					7.009	0.031
Yes	371	105(28.3)	225(60.6)	41(11.1)		
No	125	47(37.6)	59(47.2)	19(15.2)		
Whether respondents participates in social activities					3.420	0.182
Yes	412	132(32.0)	234(56.8)	46(11.2)		
No	84	20(23.8)	50(59.5)	14(16.7)		
Does respondents take health products					19.671	<0.001
Yes	115	18(15.7)	75(65.2)	22(19.1)		
No	381	134(35.2)	209(54.9)	38(10.0)		
Can respondents have at least 6 h of sleep per day					3.741	0.155
Yes	182	63(34.6)	94(51.6)	25(13.7)		
No	314	89(28.3)	190(60.5)	35(11.1)		

**Table 4:** Comparison of self-rated health of young-old comorbidities female patients with different data.

Variables	*N*	Good self-rated health [*n*(%)]	Average self-rated health [*n*(%)]	Poor self-rated health [n(%)]		*P-*value
Exercise intensity type					44.002	<0.001
Vigorous-intensity	234	132(56.4)	89(38.0)	13(5.6)		
Moderate-intensity	79	33(41.8)	32(40.5)	14(17.7)		
Light-intensity	156	48(30.8)	67(42.9)	41(26.3)		
Age					4.519	0.340
60–64	196	85(43.4)	79(40.3)	32(16.3)		
65–69	235	105(44.7)	97(41.3)	33(14.0)		
70–74	38	23(60.5)	12(31.6)	3(7.9)		
BMI					29.408	<0.001
Low weight	27	6(22.2)	18(66.7)	3(11.1)		
Normal weight	313	125(39.9)	137(43.8)	51(16.3)		
Overweight	108	69(63.9)	27(25.0)	12(11.1)		
Obesity	21	13(61.9)	6(28.6)	2(9.5)		
Comorbidity duration					29.194	<0.001
Less than 6 years	161	53(32.9)	89(55.3)	19(11.8)		
6–10 years	250	130(52.0)	75(30.0)	45(18.0)		
More than 10 years	58	30(51.7)	24(41.4)	4(6.9)		
Household registration types					7.435	0.023
Urban registration	409	195(47.7)	159(38.9)	55(13.4)		
Rural registration	60	18(30.0)	29(48.3)	13(21.7)		
Education level					9.129	0.165
Junior high school and below	124	46(37.1)	58(46.8)	20(16.1)		
High school/Polytechnic school	140	74(52.9)	52(37.1)	14(10.0)		
Junior college	242	39(42.9)	35(38.5)	17(18.7)		
College and above	220	54(47.4)	43(37.7)	17(14.9)		
Per capita annual income					12.715	0.012
Less than 30,000 yuan	78	24(30.8)	40(51.3)	14(17.9)		
30,000–50,000 yuan	161	77(47.8)	68(42.2)	16(9.9)		
More than 50,000 yuan	230	112(48.7)	80(34.8)	38(16.5)		
Living conditions					10.602	0.025
Living alone	13	5(38.5)	6(46.2)	2(15.4)		
Living with spouse	373	183(49.1)	139(37.3)	51(13.7)		
Living with children	83	25(30.1)	43(51.8)	15(18.1)		
Marital status					6.327	0.107
Married	451	208(46.1)	180(39.9)	63(14.0%)		
Widowed	17	4(23.5)	8(47.1)	5(29.4)		
Separated/Divorced	1	1(100.0)	0(0.0)	0(0.0)		
Will family members supervise exercise					21.379	<0.001
Yes	371	181(48.8)	151(40.7)	39(10.5)		
No	98	32(32.7)	37(37.8)	29(29.6)		
Whether respondents participates in social activities					31.777	<0.001
Yes	420	205(48.8)	167(39.8)	48(11.4)		
No	49	8(16.3)	21(42.9)	20(40.8)		
Does respondents take health products					35.020	<0.001
Yes	117	36(30.8)	44(37.6)	37(31.6)		
No	352	177(50.3)	144(40.9)	31(8.8)		
Can respondents have at least 6 h of sleep per day					58.629	<0.001
Yes	419	139(61.0)	78(34.2)	11(4.8)		
No	370	74(30.7)	110(45.6)	57(23.7)		

### 3.2. Gender, exercise intensity and self-rated of health

Using the self-rated of health among young-old patients with chronic comorbidities as the dependent variable and the statistically significant indicators in Table 2 as the independent variable, the model used the forward step method for an unordered multivariate logistic regression analysis. As shown in Table 5, compared with young-old comorbidities patients with poor self-rated health status, young-old patients who had been suffering from chronic comorbidities duration for less than 6 years (OR = 2.475, 95% CI: 1.292–4.743, *p* < 0.05) and engaged in vigorous-intensity exercise (OR = 4.368, 95% CI: 2.491–7.661, *p* < 0.05) were more likely to have better self-rated health status; Young-old patients who took health supplements were less likely to have a good self-rated health status (OR = 0.215, 95% CI: 0.132–0.351, *p* < 0.05); young-old male (OR = 1.926, 95% CI: 1.250–2.969, *p* < 0.05), with comorbidities duration less than 6 years (OR = 2.660, 95% CI: 1.468–4.818, *p* < 0.05), having family members’ supervision on exercise (OR = 2.298, 95% CI: 1.428–3.699, *p* < 0.05) and doing vigorous-intensity exercise (OR = 2.422, 95% CI: 1.406–4.173, *p* < 0.05) were more likely to have average self-rated health status; young-old patients who lived alone (OR = 0.351, 95% CI: 0.149–0.825, *p* < 0.05) and took health supplements (OR = 0.342, 95% CI: 0.220–0.532, *p* < 0.05) were less likely to have an average self-rated of their health status.

**Table 5:** Random multivariate logistic regression analysis of the factors influencing self-rated health status in young-old comorbidities patients.

Influencing factors	Good self-rated health status			Average self-rated health status		
	OR	(95%CI)	*P*	OR	(95%CI)	*P*
Intercept			0.689			0.259
Exercise intensity type: Light-intensity						
Vigorous-intensity	4.368	(2.491, 7.661)	<0.001	2.422	(1.406, 4.173)	0.001
Moderate-intensity	1.594	(0.887, 2.865)	0.119	0.995	(0.579, 1.709)	0.985
Gender: Female						
Male	1.021	(0.649, 1.606)	0.929	1.926	(1.250, 2.969)	0.003
BMI: Obesity						
Low weight	0.659	(0.171, 2.545)	0.545	0.702	(0.204, 2.415)	0.575
Normal weight	0.912	(0.322, 2.582)	0.862	0.889	(0.333, 2.371)	0.814
Overweight	1.220	(0.412, 3.611)	0.719	0.594	(0.211, 1.672)	0.324
Comorbidity duration: More than 10 years						
Less than 6 years	2.475	(1.292, 4.743)	0.006	2.660	(1.468, 4.818)	0.001
6–10 years	1.552	(0.849, 2.836)	0.153	0.965	(0.553, 1.686)	0.901
Living conditions: Living with children						
Living alone	0.456	(0.169, 1.232)	0.122	0.351	(0.149, 0.825)	0.016
Living with spouse	1.459	(0.797, 2.671)	0.221	0.644	(0.372, 1.114)	0.115
Will family members supervise exercise: No						
Yes	1.325	(0. 802, 2.188)	0.272	2.298	(1.428, 3.699)	0.001
Does respondents take health products: No						
Yes	0.215	(0.132, 0.351)	<0.001	0.342	(0.220, 0.532)	<0.001
Can respondents have at least 6 h of sleep per day: No						
Yes	1.631	(0.977, 2.722)	0.062	1.048	(0.640, 1.714)	0.853

Randomized multivariate logistic regression analysis was conducted on male and female patients, and the results are shown in Tables 6, 7. With young-old comorbidities male patients with poor self-rated health status as the control group, young-old male patients who had been suffering from chronic comorbidities for less than 6 years (OR = 6.167, 95% CI: 2.527–15.050, *p* < 0.05), 6–10 years (OR = 5.157, 95% CI: 2.137–12.444, *p* < 0.05), and engaged in vigorous-intensity exercise (OR = 2.924, 95% CI: 1.266–6.751, *p* < 0.05) were more likely to have better self-rated health status; Young-old male patients with chronic comorbidities who took health supplements were less likely to have a good self-rated health status (OR = 0.206, 95% CI: 0.093–0.455, *p* < 0.05); Young-old male patients with chronic comorbidities who had been suffering from chronic comorbidities diagnosed for less than 6 years were more likely to have an average self-rated health status (OR = 3.602, 95% CI: 1.736–7.476, p < 0.05); young-old male patients with chronic comorbidities who lived alone (OR = 0.273, 95% CI: 0.093–0.801, *p* < 0.05) and took health supplements (OR = 0.482, 95% CI: 0.251–0.927, *p* < 0.05) were less likely to have an average self-rated of their health status.

**Table 6:** Random multivariate logistic regression analysis of the factors influencing self-rated health status in young-old comorbidities male patients.

Influencing factors	Good self-rated health status			Average self-rated health status		
	OR	(95%CI)	*P*	OR	(95%CI)	*P*
Intercept			0.531			0.071
Exercise intensity type: Light-intensity						
Vigorous-intensity	2.924	(1.266, 6.751)	0.012	1.305	(0.592, 2.875)	0.509
Moderate-intensity	0.672	(0.285, 2.584)	0.363	0.627	(0.301, 1.306)	0.212
BMI: Obesity						
Low weight	0.924	(0.148, 5.733)	0.933	0.361	(0.075, 1.739)	0.204
Normal weight	1.623	(0.367, 7.170)	0.523	0.975	(0.277, 3.432)	0.968
Overweight	1.631	(0.363, 7.332)	0.523	0.500	(0.138, 1.805)	0.290
Comorbidity duration: More than 10 years						
Less than 6 years	6.167	(2.527, 15.050)	<0.001	3.602	(0.169, 2.889)	0.001
6–10 years	5.157	(2.137, 12.444)	<0.001	1.995	(0.044, 0.697)	0.065
Living conditions: Living with children						
Living alone	0.297	(0.079, 1.118)	0.073	0.273	(0.093, 0.801)	0.018
Living with spouse	1.129	(0.437, 2.915)	0.802	0.535	(0.231, 1.235)	0.143
Will family members supervise exercise: No						
Yes	0.881	(0.422, 1.841)	0.736	1.862	(0.945, 3.667)	0.072
Does respondents take health products: No						
Yes	0.881	(0.093, 0.455)	<0.001	0.482	(0.251, 0.927)	0.029

**Table 7:** Random multivariate logistic regression analysis of the factors influencing self-rated health status in young-old comorbidities female patients.

Influencing factors	Good self-rated health status			Average self-rated health status		
	OR	(95%CI)	*P*	OR	(95%CI)	*P*
Intercept			0.461			0.897
Exercise intensity type: Light-intensity						
Vigorous-intensity	4.232	(1.869, 9.853)	0.001	3.077	(1.379, 6.870)	0.006
Moderate-intensity	4.555	(1.825, 11.368)	0.001	2.179	(0.905, 5.245)	0.082
BMI: Obesity						
Low weight	0.896	(0.096, 8.402)	0.924	3.749	(0.420, 33.449)	0.237
Normal weight	0.884	(0.164, 4.765)	0.886	1.911	(0.326, 11.202)	0.473
Overweight	1.378	(0.238, 7.978)	0.721	1.307	(0.205, 8.330)	0.777
Comorbidity duration: More than 10 years						
Less than 6 years	0.441	(0.104, 1.868)	0.266	0.698	(0.169, 2.889)	0.620
6–10 years	0.211	(0.849, 2.836)	0.028	0.176	(0.044, 0.697)	0.013
Will family members supervise exercise: No						
Yes	2.460	(1.143, 5.291)	0.021	2.410	(1.079, 5.383)	0.032
Whether respondents participates in social activities: No						
Yes	6.173	(2.285, 16.678)	<0.001	2.410	(1.079, 5.383)	<0.001
Does respondents take health products: No						
Yes	0.227	(0.109, 0.476)	<0.001	0.225	(0.111, 0.457)	<0.001
Can respondents have at least 6 h of sleep per day: No						
Yes	4.314	(1.895, 9.819)	<0.001	2.222	(0.980, 5.040)	0.056

With young-old female chronic comorbidities with poor self-rated health status as the control, young-old female patients who were supervised by their family to exercise (OR = 2.460, 95% CI: 1.143–5.291, *p* < 0.05), participated in social activities (OR = 6.173, 95% CI: 2.285–16.678, *p* < 0.05), had at least 6 h of sleep per day (OR = 4.314, 95% CI: 1.895–9.819, *p* < 0.05), and engaged in vigorous-intensity (OR = 4.232, 95% CI: 1.869–9.583, *p* < 0.05) and moderate-intensity (OR = 4.555, 95% CI: 1.825–11.368, *p* < 0.05) exercise were more likely to have better self-rated health status; young-old female patients with chronic comorbidities who had been suffering from chronic comorbidities for 6–10 years (OR = 0.211, 95% CI: 0.053–0.844, *p* < 0.05) and had taken health supplements (OR = 0.227, 95% CI: 0.109–0.476, *p* < 0.05) were less likely to have a good self-rated health status; young-old female patients with chronic comorbidities who were supervised by their families to exercise (OR = 2.796, 95% CI: 1.330–5.875, *p* < 0.05), participated in social activities (OR = 2.410, 95% CI: 1.079–5.383, *p* < 0.05), and engaged in vigorous-intensity exercise (OR = 3.077, 95% CI: 1.379–6.870, *p* < 0.05) were more likely to have an average self-rated health status; young-old female patients with chronic comorbidities who had been suffering from chronic comorbidities for 6–10 years (OR = 0.176, 95% CI: 0.044–0.697, *p* < 0.05) and have took health supplements (OR = 0.225, 95% CI: 0.111–0.457, *p* < 0.05) were less likely to have an average self-rated health status.

## 4. Discussion

We found that young-old patients with chronic comorbidities who engaged in vigorous-intensity exercise tended to have better self-rated health. This was similar to the research results of Norton et al. (35) and Marques et al. (36), which indicated the help of exercise in cardiovascular and metabolic diseases in older comorbidities patients. Vigorous-intensity exercise can promote the adaptability and improvement of body. Although older patients with chronic comorbidities may face health problems, moderate-intensity exercise and vigorous-intensity exercise can improve muscle strength, cardiovascular and metabolic function (37), having a positive effect on the health of older patients, helping them prevent the occurrence of chronic diseases, and reducing the harm of these diseases to their body. These improvements may make patients feel more energetic and healthy. Previous studies have shown that moderate exercise has a predictive effect on improving health and quality of life (38). Individuals who actively participate in sports activities are able to maintain or improve their physical abilities and better complete daily tasks in daily life (39), leading respondents to make better health self-rated health status. Meanwhile, vigorous-intensity exercise may have a positive effect on the endocrine system, such as promoting the release of neurotransmitters such as endorphins and dopamine, thereby improving emotional and psychological states (30). Exercise therapy is a treatment method often used to improve the physical and mental health of older people without any side effects (31). Vigorous-intensity exercise can significantly help the treatment results, health, metabolism and exercise ability of some older people with chronic comorbidity (such as hypertension, cardiovascular disease, type 2 diabetes, clinical depression, osteoporosis, muscle weakness, etc.) (40). By persisting in vigorous-intensity exercise, not only can the risk of depression in older chronic comorbidities patients be reduced (41), but also their confidence in their own abilities (42) can be improved, believing that they can make positive changes in their physical health. These factors can enable respondents to more positively evaluate their health status.

Not all older comorbidities patients are suitable for and can adapt to vigorous-intensity exercise levels. Studies by Endeshaw and Goldstein (43) and Lam et al. (44) have shown that mild and moderate-intensity exercise can help older people maintain health. As long as the exercise intensity is appropriate and sufficient, older patients will be healthier and have a lower risk of cardiovascular disease (45). And studies have shown that exercise have different effects on the health status of older men and women (46), with vigorous-intensity exercise having a stronger adverse effect on women’s health than men (31). In this study, we found that young-old male patients with chronic comorbidities needed to engage in vigorous-intensity exercise in order to have a positive effect on their self-rated health; Young-old female patients with chronic comorbidities who engaged in vigorous-intensity and moderate-intensity exercise can have a positive effect on their self-rated of health, which was similar to the research findings of Marques et al. (47). It may be due to physiological differences between men and women, including hormone levels, body composition, and muscle mass. These physiological differences may lead to different responses to exercise. Men typically have more muscle mass and higher metabolic rate (48), therefore requiring higher intensity exercise to produce significant effects. And there are also differences in the types of comorbidities between males and females. For example, female comorbidities patients are more likely to suffer from osteoporosis, rheumatism, etc. (49). Vigorous-intensity exercise is not conducive to the recovery of the condition and may lead to worsening symptoms. The results of this study indicated that compared to men, women only needed to engage in moderate-intensity exercise to positively affect their self-rated health, which could avoid the risk of injury caused by vigorous-intensity exercise (50). Additionally, compared to vigorous-intensity exercise, moderate-intensity exercise is more accepted by older patients and can be persisted for a long time, which contributes to the long-term beneficial effect of exercise on the health of comorbidities patients (51).

Regular exercise is crucial for a healthy aging population (15). At present, many communities have adopted a multidisciplinary and comprehensive approach to the management of older patients’ chronic diseases, including nutrition, exercise, cognition, emotions, and so on. For older comorbidities patients, a reasonable exercise method is mixed exercise, which combines aerobic exercise with resistance exercise. This can strengthen the quality of bones and muscles of older patients, significantly reduce the risk of chronic diseases, promote health, and improve the quality of daily life (52). Although the benefits of exercise on the health of older patients with chronic comorbidities are significant, the frequency of exercise will decrease with age (53). Due to various influencing factors, it is difficult for older people to achieve the recommended exercise intensity. Therefore, integrating comprehensive interventions in community health services such as exercise and chronic disease management can improve the health status of older people with chronic diseases in the community. Managing and controlling symptoms related to chronic diseases can optimize the health management of older comorbidities patients and significantly increase their level of exercise participation. When managing chronic diseases among older people in the community through exercise, it is important to pay attention to their exercise compliance management. The key is to integrate their lifestyle with exercise. Communities can also regularly organize sports that are suitable for older people. The most reasonable and effective way may be to customize personalized exercise plans for older people in the community based on their lifestyle and physical health status (54), Based on the results of this study, different intensities of exercise can be recommended for older people of different genders. For example, men should recommend more vigorous-intensity aerobic exercise, while women should recommend moderate-intensity aerobic and resistance training (55). The specific exercise plan (including the type and frequency of exercise, etc.) needs to be designed by healthcare professionals such as professional physical therapists, exercise physiologists, or fitness coaches based on each individual’s physical condition, type and quantity of chronic diseases, and other specific circumstances. At the same time, attention should also be paid to safety protection measures during the exercise process, such as the use of safety facilities or specialized guidance, to avoid other damages to their body during the exercise process.

We observed an interesting result in whether to participate in social activities and self-rated of health. Young-old female patients with chronic comorbidities who were urged by their families to exercise and participate in social activities were more inclined to have better self-rated health, while this result was not significant among males. It is possible that older women have a higher probability of widowhood than men (56), and their own economic and social abilities are weaker than men, making them more dependent on their children and other relatives in the family for care. Moreover, the care of children and other relatives plays an important role in the physical and mental health of older people (57). Previous studies have shown that social participation is associated with positive health behaviors among older people (58), and older people who frequently participate in social activities are more likely to engage in vigorous-intensity exercise; less social participation significantly positively affects older people’s lack of physical activity, self-rated health, and low quality of life (59). The supervision, support, and assistance of family members can also encourage older comorbidities patients to actively engage in self-care (60), improve satisfaction with health and subjective well-being, make them feel more capable and confident in overcoming problems encountered, achieve their goals, improve their self-efficacy in exercise, and actively engage in beneficial behaviors (such as exercise), thereby positively affecting their self-rated of health outcomes. Based on this, community support networks can be established to encourage older patients with comorbidities to participate in community activities and maintain contact with family and friends, alleviate loneliness and depression, improve self-efficacy, promote exercise, and thereby reduce the risk of developing more diseases and improve self-rated health (61). However, this study mainly focuses on the relationship between gender, exercise intensity, and self-rated health. The effect of participation in social activities on self-rated health requires more detailed research in the future.

### 4.1. Limitations

Firstly, due to the data source of this study being a structured questionnaire, several items in the questionnaire lacked standardized measurement methods. Only several items were flawed and the effectiveness and feasibility of this questionnaire survey were proven, nevertheless, it might still result in possible differences in measurement results between different time points and respondents. Moreover, the expression of individual questions might not be clear and clear enough, causing misunderstandings or difficulties in selection among respondents. This might guide participants to answer in certain specific directions, resulting in report bias and ultimately inaccurate data collection, affecting the reliability of research results.

Secondly, the outcome variable of this study was the respondents’ self-rated health status, which was based on an individual’s subjective perspective and was therefore influenced by their culture, social experience, and emotional state. During the evaluation process, subjects were prone to memory bias, and the evaluation results were subjective and relative, leading to distortion in health rated. Individual self-rated of health might also be influenced by social expectations. Individuals might tend to answer that meet social expectations to avoid being perceived as “unhealthy” or different from others. This might lead to distortion in the evaluation results. In addition, the independent variable of this study, the type of exercise intensity, was determined by researchers based on previous studies and the definition in the guidelines. Considering the discrete duration variables of exercise and a large amount of missing data, we were unable to calculate the duration of different activity intensity levels. Moreover, this study did not clearly define which specific exercises should be included in each exercise intensity, which was detrimental to the practical application of the results. There were many influencing factors on the self-rated of health among older comorbidities patients, but due to the limited control variables in this study, detailed analysis was not possible and further research was needed. The above possibilities might lead to a lack of objectivity in the results of this study, limiting the promotion and comparison of different research results. If we consider the above information, we may have a better understanding of the relationship between exercise intensity and the health of older comorbidities patients, and the guidance provided by the research results may be more useful for policies and intervention measures. This indicated that future research required standardized research methods and tools. These studies will guide actions aimed at promoting physical exercise, quality of life, and other health factors in older comorbidities patients.

Finally, as this study was cross-sectional, the results of this study only indicated that older patients with chronic comorbidities who engaged in vigorous-intensity to moderate-intensity exercise have relatively better self-rated of health status. The changes in outcomes was not tracked over time in the study, so the clear evidence of causal relationships could not be provided. This also made it difficult to draw conclusions on the relationship between different exercise intensities and health conditions.

## 5. Conclusion

In summary, representative data from Guangdong Province indicated that vigorous-intensity exercise had a significant positive effect on the self-rated health of young-old patients with chronic comorbidities, provided that their physical condition permits. In terms of gender, young-old male patients with chronic comorbidities who engaged in vigorous-intensity exercise are more likely to considerate their health as good; for female patients, both vigorous-intensity and moderate-intensity exercise could improve their self-rated health status. This finding suggests that exercise should be integrated into the management of older chronic comorbidities patients. The exercise style, intensity type, and frequency of older comorbidities patients should be differentiated and determined according to gender to promote their health. It is recommended to conduct a follow-up study to verify the causal relationship between different exercise intensities and the health of older comorbidities patients, and more research is needed to determine the specific exercise patterns, times, and frequencies corresponding to different intensities of exercise, as well as the specific types of exercise corresponding to which chronic diseases are beneficial.

## Data availability statement

The raw data supporting the conclusions of this article will be made available by the authors, without undue reservation.

## Author contributions

LL: Writing – original draft, Writing – review & editing. FD: Writing – review & editing. DZ: Writing – review & editing.
